# Conformational gating of DNA conductance

**DOI:** 10.1038/ncomms9870

**Published:** 2015-12-09

**Authors:** Juan Manuel Artés, Yuanhui Li, Jianqing Qi, M. P. Anantram, Joshua Hihath

**Affiliations:** 1 Department of Electrical and Computer Engineering, University of California Davis, One Shields Avenue, Davis, Califorina 95616, USA; 2 Department of Electrical Engineering, University of Washington, 185 Stevens Way, Seattle, Washington 98195-2500, USA

## Abstract

DNA is a promising molecule for applications in molecular electronics because of its unique electronic and self-assembly properties. Here we report that the conductance of DNA duplexes increases by approximately one order of magnitude when its conformation is changed from the B-form to the A-form. This large conductance increase is fully reversible, and by controlling the chemical environment, the conductance can be repeatedly switched between the two values. The conductance of the two conformations displays weak length dependencies, as is expected for guanine-rich sequences, and can be fit with a coherence-corrected hopping model. These results are supported by *ab initio* electronic structure calculations that indicate that the highest occupied molecular orbital is more disperse in the A-form DNA case. These results demonstrate that DNA can behave as a promising molecular switch for molecular electronics applications and also provide additional insights into the huge dispersion of DNA conductance values found in the literature.

## Abstract (web summary)

DNA could find a role in molecular electronics. Here, the authors show that the conductance of DNA can be reversibly changed by an order of magnitude when its conformation is changed from one form to another by controlling its chemical environment.

## Graphical abstract

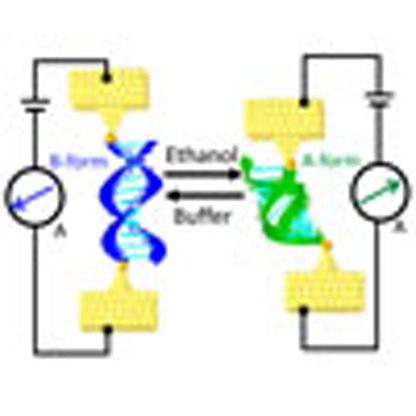

Beyond its notable role as the primary carrier of genetic information, DNA has recently emerged as an important material for nanotechnology^1^,^2^,^3^,^4^. Its exceptional self-assembly capabilities have enabled the development of nanostructured systems with unparalleled precision at this scale^5^,^6^,^7^,^8^. In addition, photochemical measurements have demonstrated long-range charge transfer in DNA^9^, and it has been suggested that these electronic properties may be involved in biological processes such as enzymatic signalling or recognition of oxidative damage^10^. The combination of these unique structural and electronic properties^1^,^2^ also make DNA a promising molecule for molecular electronics applications^11^,^12^. As such, the electronic properties of DNA have received considerable attention in recent years, and because of both the biological and technological significance of this molecule, many experimental and theoretical studies have focused on understanding charge transport in DNA^13^. Although considerable progress has been made in understanding charge transfer processes through DNA photochemically^14^,^15^,^16^ and electrochemically^10^,^14^, direct DNA conductance measurements have resulted in reported values that span many orders of magnitude^13^,^17^. More recently, single-molecule conductance measurements using break junction-based approaches^18^ that are capable of performing large numbers of single-molecule experiments have been used to obtain reproducible conductance values for short double-stranded DNA (dsDNA) sequences^19^,^20^,^21^,^22^,^23^,^24^,^25^,^26^. These measurements have demonstrated that the conductance of dsDNA molecules in solution are sensitive to length and sequence^19^, single-nucleotide polymorphisms^20^ and even epigenetic effects like the methylation of cytosines^21^.

On the theoretical front, several recent efforts have demonstrated the importance of the duplex conformation on the transport properties^13^,^17^,^27^,^28^,^29^. Oligonucleotides can adopt different conformations in solution depending on the sequence and environment^30^,^31^, and the best characterized form of DNA is the right-handed, B-form, helix. This form is normally adopted by dsDNA in physiological conditions^32^, but DNA can also adopt other structures, including a more compact helix known as the A-form^30^. The A-form is the prototypical structure for double-stranded RNA and DNA:RNA hybrids and can be induced in dsDNA by low hydration conditions. It is also a right-handed helix, but has a shorter rise per base pair (bp) than the B-form (0.23 versus 0.34 nm bp^−1^), and it also plays important roles in biological processes, such as transcription^30^. The fact that DNA can adopt different conformations has been suggested as one of the underlying reasons for the large variation in experimental DNA conductance values^13^,^17^,^27^,^33^. In addition, although the observation that the base alignment is worse in A-form than in B-form led to the prediction that the A-form is insulating^17^, recent photochemical studies using A-form DNA:RNA duplexes in solution reported an efficient charge transfer process^34^.

Here we report that the conductance of DNA duplexes increases by approximately one order of magnitude when its conformation is changed from B-form to A-form. This large and unexpected conductance increase is fully reversible, and by controlling the environment, the conductance can be repeatedly switched between the two values. Length-dependent conductance studies of the two conformations suggest that neither tunnelling nor simple hopping dominate the charge transport processes in these guanine-rich sequences, and a coherent correction to the hopping model must be included to fit the experimental results. In addition, *ab initio* electronic structure calculations coupled with calculations of the electronic density of states (DoS) of the two conformations corroborate the experimental finding that the A-form should in fact have a higher conductance value than the B-form. Furthermore, by directly demonstrating that conformation plays a large and important role in the transport properties, these results also help rationalize the large dispersion of DNA conductance values found in the literature where the DNA may be in aqueous^19^ or ambient^35^ conditions, or on an inorganic substrate^36^.

## Results

### Structural modulation of conductance in DNA

**Figure 1:**
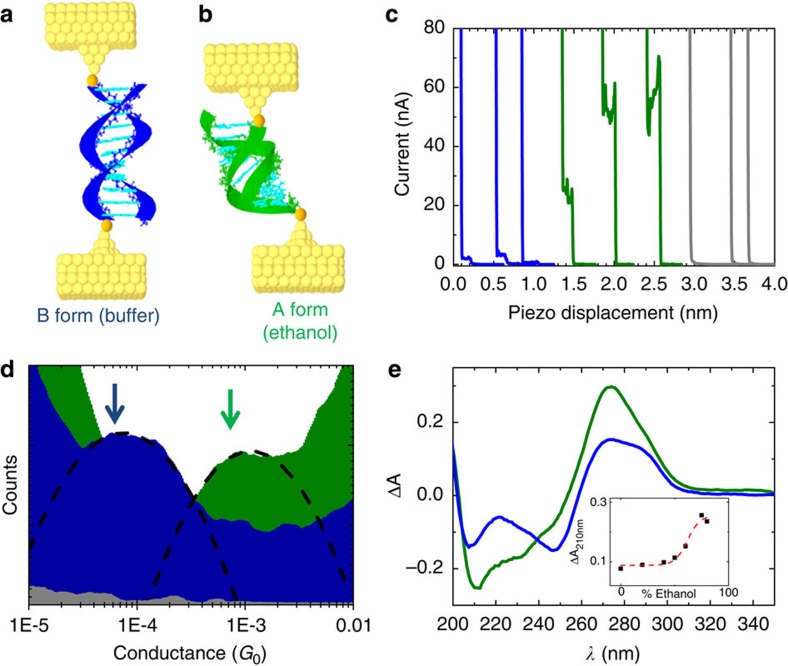
Structure-conductance modulation in 11-mer (NH_2_-5′-CCCGCGCGCCC-3′-NH_2_) dsDNA. Schemes showing (**a**) B-form (blue) and (**b**) A-form (green) dsDNA bound to Au electrodes. The linkers to the electrodes (either amines or thiols) are shown in orange. (**c**) Representative current versus distance curves measured during electrode separation in buffer (blue) and 80% ethanol (green) solutions containing dsDNA, and in the absence of the molecule (grey) (curves offset horizontally for clarity). (**d**) Logarithmic conductance histograms obtained from thousands of current versus distance traces for B-form DNA in buffer (blue), A-form DNA in ethanol (green) and a control experiment without DNA present (grey), the control histogram is increased by 20 times for clarity. Dashed lines are Gaussian fittings. *G*_0_ is the quantum conductance. (**e**) Circular dichroism spectra (differential absorbance versus wavelength) for dsDNA in solution in the same conditions described in **b**, demonstrating a clear change from B-form to A-form. Inset shows a titration of the intensity at 210 nm versus increasing concentrations of ethanol in the solution.

In this report, we directly demonstrate that the conductance of DNA duplexes is greatly affected by the conformation of DNA by reversibly switching DNA between the B-form and the A-form in solution. We began by measuring the conductance of diamine functionalized dsDNA junctions (5′-CCCGCGCGCCC-3′ plus complementary strand, see Supplementary Tables 1 and 2) using the scanning tunnelling microscope (STM)-break junction approach^18^. Experiments were performed in phosphate buffer, where the oligonucleotide adopts the B-form (Fig. 1a), and in an 80% ethanol solution, where dsDNA adopts the A-form structure (Fig. 1b)^30^. Figure 1c shows typical current versus distance traces obtained from break junction experiments containing dsDNA with amine linkers in buffer (blue curves), in 80% ethanol (green curves), and for experiments without DNA present (grey curves). Control experiments in solutions not containing dsDNA, or without a linker present (amino or thiol ending groups), show no significant plateaus in the exponential trace (grey curves in Fig.1c and histograms in Supplementary Fig. 1). Additional control measurements performed with diamine-functionalized single-stranded DNA under the same conditions do not show steps in this conductance range (see Supplementary Fig. 2 for details), indicating that charge transport in dsDNA depends on a subtle interplay between π-stacking and H-bonding in the bases^37^. Hundreds of traces similar to those shown in Fig. 1c are added to a conductance histogram to determine the most probable conductance of a single-molecule junction (Fig. 1d). The average conductance value measured in aqueous solution for dsDNA (B-form) is within the range of previously reported conductance values for short GC-rich dsDNA in solution^19^. To explore the possibility of multiple conductance peaks, we measured the same sequence with different current amplifiers and applied bias voltages (see Supplementary Fig. 3 for details) and a single prominent peak consistent with Fig. 1d was obtained in all cases. In addition, to ensure that the forces applied during the pulling cycle of the break-junction process were not responsible for the conductance differences, we performed conductance measurements on spontaneously formed DNA junctions with a constant distance between the STM tip and substrate^38^. The conductance value obtained using this method agrees well with the one obtained by the STM-break junction approach (see Supplementary Fig. 4 for details), indicating that the strain induced during the break-junction process does not significantly affect the measured conductance value on the conductance plateau, in agreement with previous reports^39^.

The average conductance value obtained for dsDNA in ethanol solutions (green histogram) is one order of magnitude higher than the one obtained for the same sequence in buffer conditions (blue histogram), see Fig. 1d and Supplementary Fig. 5 for details. We confirmed that a B- to A-form transition occurs when ethanol is added using circular dichroism spectroscopy (see Fig. 1e and Supplementary Fig. 6 for details), and a clear transition from B-form to A-form occurs when the ethanol concentration is above 60%. Briefly, the differential absorbance at 210 nm shows an intense negative peak when the molecule is in A-form^30^. In addition, we note that these sequences are stable at room temperature, Supplementary Table 2 in the supporting information shows the measured sequences along with calculated melting temperatures (*T*_m_) for both B-form and A-form oligonucleotides.

In an alternative approach to measuring the conductance, we recorded the current as a function of an applied voltage sweep once the junctions were detected to obtain *I–V* characteristics (Supplementary Fig. 7). Interestingly, the *I–V* characteristics obtained are linear in the voltage range studied, and do not indicate a threshold voltage below 1 V for the onset of current flow. These results confirm that the transport is ohmic in aqueous solution at low biases and room temperature^13^,^26^, and the slope of the *I–V* characteristics yields a conductance value that is consistent with the values obtained from the histograms. Both the conductance and *I–V* characteristics demonstrate that the dsDNA duplex in A-form has a higher conductance value than in the B-form. This observation is contrary to expectations, because although the rise per base is smaller, the bases are actually tilted by ∼20° (versus the backbone) in the A-form. This fact suggests that the π-orbital coupling between bases should be significantly weaker, and thus the conductance should be lower in the A-form, which has been predicted previously^17^.

### Length dependence of B and A-form conductance

**Figure 2:**
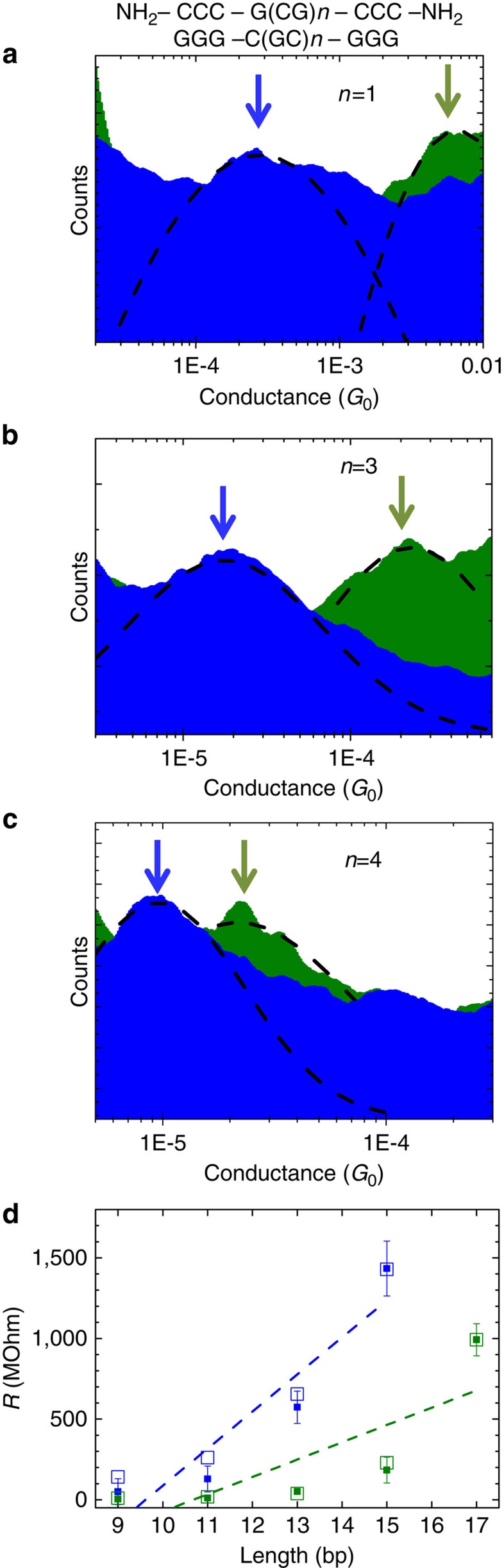
Length dependence of the conductance of different conformations. Length dependence of the conductance B-form (blue) and A-form (green) diamine-functionalized dsDNA. Logarithmic conductance histograms for (**a**) 9-mer, (**b**) 13-mer and (**c**) 15-mer dsDNA. Dashed lines are Gaussian fits to obtain the most probable conductance. (**d**) Resistance values obtained from single-molecule conductance experiments are shown versus oligonucleotide length for B-form (blue) and for A-form (green) as solid symbols. Dashed lines are linear fittings corresponding to a hopping process with each guanine base acting as an independent hopping site. Void symbols are predicted values for a hopping process with a coherent correction^44^ (see Supplementary Methods for details). Error bars represent the standard error of the mean with *N*=3 measurements.

To provide additional insights into the transport properties of the different conformations, we measured the length dependence of the conductance of a series of poly-GC sequences for both conformations as shown in Fig. 2a–c. Blue and green histograms correspond to experiments performed in phosphate buffer and 80% ethanol solution, respectively. The conductance measured in the ethanol solution is always ∼10 times higher than the one measured in buffer and confirms that the A-form DNA conductance is higher than the B-form conductance for the same number of bp. The conductance values of sequences ranging from 9 to 17 bp in length are plotted on a semi-logarithmic conductance versus distance scale in Supplementary Fig. 8, and the slopes of these plots can be used to obtain the distance decay factor (*β*), which is typically characteristic of a molecular family and can provide insights into the charge transport mechanism^40^. Here we obtain apparent *β*-values of 0.18 and 0.33 Å^−1^, for the B-form and A-form, respectively. Although these values are different for the two conformations, both are within the range typically attributed to a multi-step hopping transport mechanism^41^.

The simplest hopping model treats each guanine base as an independent hopping site, and results in a resistance (*R*) that is proportional to the number (*N*) of hopping sites (bases)^42^,^43^. However, as can be seen in Fig. 2d, there is not a simple linear relationship between *R* and *N* for these data. These results indicate that even though the apparent *β*-value is consistent with a multi-step hopping mechanism, the simple hopping model does not apply, and this indicates that some coherent process may be contributing to the charge transport process through these oligonucleotides^44^,^45^. To examine this possibility, a coherent correction term was added to the hopping formalism to fit the length-dependent conductance data, as was described previously^44^,^45^,^46^. Using this coherence-corrected formalism, the predicted resistance values (open squares in Fig. 2d) are closer to the experimental results, and yield coherence lengths of ∼4 and ∼5 nm for the B-form and A-form, respectively. These long coherence lengths indicate that the delocalization lengths may be significantly longer than previously assumed^44^,^45^, and that the delocalization may be longer in the A-form than the B-form. Fitting the data with these three different conduction models suggests that the dominant transport mechanism in these sequences is actually neither simply tunnelling (because of the low β-values) nor fully incoherent hopping (because of the poor fitting), but is in fact dominated by a process that has a large coherent component but still yields a high conductance value over these length scales (up to ∼5 nm).

### Reversible conformational gating of DNA conductance

**Figure 3:**
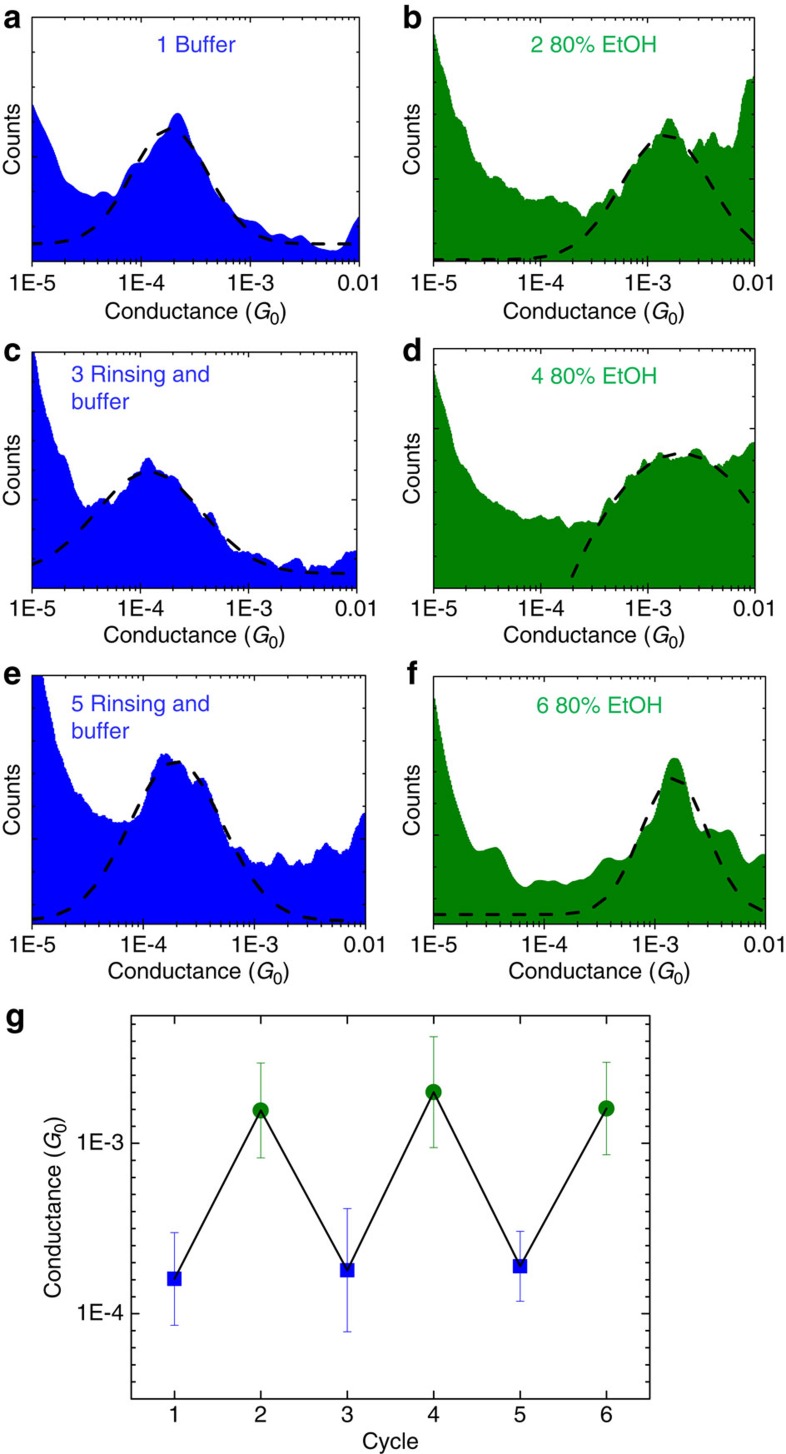
Structure conductance control in dithiol-modified 11-mer dsDNA. Logarithmic conductance histograms for experiments performed in (**a**) buffer, (**b**) 80% ethanol, (**c**) rinsing and measuring in buffer, (**d**) 80% ethanol, (**e**) rinsing and measuring in buffer again and finally (**f**) measuring in 80% ethanol again. Dashed lines are Gaussian fittings to find the most probable conductance. (**g**) Conductance values obtained upon multiple exchanges of solvent on a single sample. Error bars represent the standard deviations obtained from the fittings. The process is completely reversible over the six cycles studied.

Now we demonstrate that this structure-conductance modulation is fully reversible. Figure 3 shows conductance histograms obtained from a sample containing dithiol-modified dsDNA during six consecutive solvent exchange cycles between the phosphate buffer and the 80% ethanol solution. Thiols were used in this case to ensure that the molecules are strongly bound to the surface, and remain there during rinsing and solvent exchange. The conductance value obtained for the dithiol-modified 11-mer dsDNA in Fig. 3 is similar to the value obtained in diamine-modified dsDNA (see Fig. 1c for details), emphasizing that this switching in conductance is not caused or influenced by the linking group. Figure 3g shows the most probable conductance value obtained from the histograms in Fig. 3a–f, corresponding to different cycles of structural switching over the same sample. Results show a fully reversible modulation of the average conductance by inducing a structural change in single dsDNA molecules.

### Electronic structure and transport calculations

**Figure 4:**
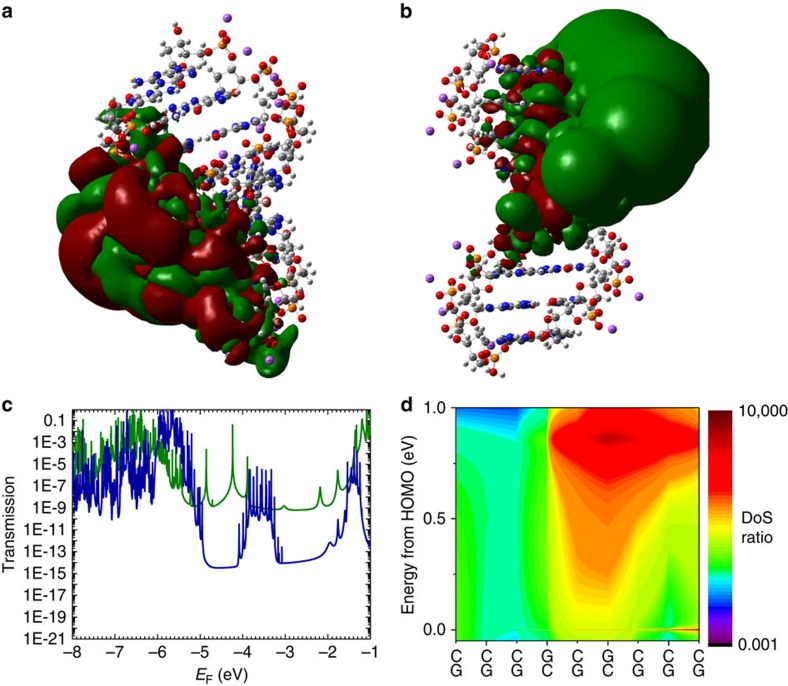
Results from *ab initio* calculations on 9-mer dsDNA. (**a**) 3D iso-surface of the HOMO orbital (iso-value=0.00002) on the oligonucleotide structures for A-form and (**b**) B-form dsDNA. (**c**) Calculated transmission probability for A-form (green) and B-form (blue) dsDNA. For most energies in the HOMO–lowest unoccupied molecular orbital (LUMO) gap, A-form transmission is orders of magnitude higher than B-form transmission. (**d**) 2D representation of the ratio of total density of states along the molecule between A-form and B-form along the different bases and for 1 eV energy range in the HOMO–LUMO gap for 9-mer. Colour scale is logarithmic going from 0.001 (black) to 10,000 (dark red), where green is 1. In the central bases of the molecule, DoS for the A-form is several orders of magnitude higher than in the B-form.

To gain an understanding of why the A-form has a higher conductance, and the reasons for the discrepancies with previous predictions about the A-form conductance^17^, we performed a series of *ab initio* electronic structure calculations. Using the results from these calculations, we analysed (i) the transmission (Fig. 4c and Supplementary Fig. 9), (ii) the highest occupied molecular orbital (HOMO) distribution (Fig. 4a,b and Supplementary Fig. 10), (iii) the projected probability density of the HOMO level along the molecular length (Supplementary Fig. 11) and (iv) the DoS (Fig. 4d and Supplementary Fig. 12). The *ab initio* calculations were performed using the B3LYP/6-31G(d,p) functional and basis set^47^.

We start by examining the transmission for each form of the molecule (see Methods section). The transmission through the DNA duplexes is a measure of the ease of propagation of electrons injected in the left-end and collected in the right-end (and vice versa). The transmission calculations are performed both in the coherent limit and with decoherence included (see Supplementary Methods for details), using the Green's function approach^48^. Figure 4c shows that the coherent transmission in the HOMO-lowest unoccupied molecular orbital (LUMO) gap is significantly higher in the A-form than in the B-form, indicating that charge propagation should be easier in the A-form than the B-form, and is consistent with our experimental results discussed above. However, the absolute transmissions in the fully coherent case are significantly lower than the experimentally obtained conductance values, verifying that simple tunnelling is not capable of explaining the results alone. Including decoherence in the transport model (see Supplementary Fig. 13 for details) makes the conductance magnitude closer to the experimental values, as one would expect from the coherence-corrected hopping model used above (Fig. 2d).

To obtain more physical insight into the reasons that the A-form has a higher conductance than the B-form, we examine the features of the HOMO energy level in both forms of the molecule. Figure 4a,b plots the HOMO iso-surface that is obtained from calculations of the 9-mer dsDNA (5′-CCCGCGCCC-3′ plus complement) when all of the bases and the backbone are included in the calculation. At an iso-value of 2 × 10^−5^, the HOMO level is distributed over 70% of the molecular length in the A-form (Fig. 4a), but only over 50% in the B-form (Fig. 4b). This result is not only specific to the 9-mer, but is consistent across the entire family of molecules studied (see Supplementary Fig. 10 for details). To present a quantitative picture of the HOMO distribution, we analysed the HOMO-level distribution for the two forms of the molecule by calculating the projected density of the HOMO-level along each strand (Supplementary Tables 3 and 4 and Supplementary Fig. 11). In agreement with the iso-surface plots in Fig. 4, these results show that the HOMO level in the A-form is more delocalized than in the B-form. These results are consistent with the longer coherence length obtained from the coherence-corrected hopping model in Fig. 2d, and suggest that a simple, nearest-neighbour hopping model is insufficient to describe the system.

The *ab initio* calculations indicate that neither tunnelling nor hopping alone are capable of explaining the experimental results. Thus, to understand the reason for the conductance difference, we focus on a more fundamental value, the DoS. Figure 4d plots the ratio of the DoS of the A-form to the B-form over the relevant energy range from 50 meV below to 1 eV above the HOMO energy level along the entire sequence of the 9-mer DNA. We observe that in the GGG triplet regions at the left and right ends of the DNA strands, the A-form and B-form DoS are similar on an average. The main difference is in the *central bridge region* with the alternating GC sequence. Here, the A-form almost always possesses a larger DoS than the B-form (by as much as 4 orders of magnitude). In addition, at the HOMO energy level, the DoS is consistently larger and more spatially diffuse in the A-form than in the B-form (see Fig. 4d for details). This observation leads us to conclude that the higher conductance of the A-form is due to the large difference in the DoS in the central bridge region.

Finally, we note that all of the analysis we have performed indicates that A-form should be higher in conductance than B-form in agreement with the experimental results, but this observation is contrary to earlier predictions^17^. To understand this discrepancy, we also examined the transmission properties of these same sequences *without* including the molecular backbone, as has been done previously (see Supplementary Fig. 9 for details). In this case, we are able to reproduce the earlier findings of a higher transmission probability in the B-form over a wide range of energies (Supplementary Fig. 9). This finding suggests that it is necessary to include the backbone in the electronic structure calculations to be able to obtain even qualitatively correct results, and that the backbone has a significant impact on conductance. From the HOMO iso-plots (Supplementary Fig. 10) and HOMO projection analysis (Supplementary Fig. 11 and Supplementary Tables 3 and 4), it is clear that including the backbone structures has a much larger impact on the distribution of the HOMO level in the A-form than in the B-form. We hypothesize that this difference occurs because the angle between the bases and the backbone in the A-form induces more changes in the charge density distribution in the bases than in the B-form, which results in a more disperse energy level, a higher conductance and a longer coherence length.

## Discussion

These results demonstrate the possibility of controlling the conductance of DNA by modulating its structure; they emphasize the necessity for controlling the local environment in order to obtain reproducible transport measurements; and because they clearly demonstrate the role of structure on the transport, they help rationalize the large dispersion in reported DNA conductance values. The effect observed here can be relevant for the design of nanodevices based on DNA, which would largely benefit from designed self-assembly techniques used with DNA origami^5^. In addition, electrical signals from structure-conductance modulation could be exploited for the electrical detection of oligonucleotides with the same sequence but different structures, opening new perspectives in the study of fundamental biological processes and novel methods for the detection of diseases.

## Methods

### Sample preparation

DNA oligonucleotides were purchased from Biosynthesis Inc., with either diamine or dithiol functionalization on both the 5′ and 3′ ends of the same strand, see Supplementary Tables 1 and 2 for details. In the case of thiolated-DNA, the thiol group was reduced using tris(2-carboxyethyl)phosphine and purified using 7K molecular weight cut-off (MWCO) desalting columns (#89882 Zeba spin desalting columns). dsDNA was obtained by hybridization of the complementary strands by heating the mixture to 80 °C in a water bath and letting it cool down to room temperature over the course of several hours.

Na_2_HPO_4_ and NaH_2_PO_4_ were purchased from Sigma-Aldrich to create 10 mM pH 7.5 phosphate buffer. Either absolute ethanol from Sigma-Aldrich or generic alcohol from EMD were used to prepare the 80% ethanol solutions, and no significant differences were observed in the experimental results. Distilled water was used for preparing all solutions.

We used a Molecular Imaging Pico-STM head connected to a modified Digital Instruments Nanoscope E controller. The break junction experiments were performed using a LabVIEW programme developed in-house.

### Single-molecule conductance measurements

We measured dsDNA conductance using the STM break-junction approach^18^. The STM tip was prepared by cutting a 0.25-mm gold wire (99.998%, Alfa-Aesar) and then coating it with Apiezon wax to minimize the exposed surface area and limit the leakage current to ∼pA levels. A Teflon STM cell was used to hold solvent in which the measurements were performed. The cell was cleaned with piranha solution and then rinsed in distilled water several times (Caution: piranha solution reacts violently with most organic materials and must be handled with extreme care). The gold substrate was prepared by electron-beam evaporation of 130 nm of gold on a freshly cleaved mica surface. Before each experiment, the substrate was briefly flame-annealed using a butane flame.

During break-junction measurements, the STM tip was repeatedly brought into and out of contact with a gold substrate with the molecule in solution, and the current was monitored as the tip was pulled away from the substrate. Steps in the current decay trace indicate the formation of Au-DNA-Au junctions. By adding thousands of current versus distance traces together, conductance histograms are obtained, which reveal the most probable conductance of the junction. These experiments were performed either in 10 mM phosphate buffer at pH 7.5 or in a solution containing 80% ethanol (v/v). Experiments were performed at 22±2 °C. Buffer solutions were filtered using 20 nm pore size filters (Anotop 25 0.02 μm, Whatman GE Healthcare Bio-Sciences), before measurements. Before the addition of DNA sequences into the cell, preliminary blank curves were collected in buffer solution and in ethanol, to ensure that no steps were obtained in the absence of the molecule in solution (see Fig. 1c,d and Supplementary Fig. 1 for details). Small volumes of dsDNA were added to the cell in order to obtain final concentrations in the μM range. We only included curves with steps in our histograms and a LabVIEW programme was used to generate the logarithmic histograms from the data processing of collected curves^49^. Briefly, the programme performs a logarithmic binning of the current versus distance traces to create a single-trace histogram, and then detects when the conductance steps yield peaks in the histogram that are higher than a certain number of counts. All curves meeting this criterion are selected and added to a semi-logarithmic histogram. Note that the use of logarithmic histograms makes the integer values of conductance corresponding to multiple molecules in the junction collapse into a single peak. The final graphs for the manuscript were smoothed using an adjacent-averaging method with a 10-point window.

Current–voltage experiments (Supplementary Fig. 7) were performed using the method described previously in ref. 50.

### Circular dichroism

Circular dichroism experiments were performed using an Olis DSM 20 Circular Dichrometer with a 5-mm path length cylindrical cell (2 ml) at 20 °C. Before every measurement with dsDNA, a blank spectrum was collected and subtracted from the experimental data. 40 μl of 150 μM dsDNA were added to the cell before spectra collection. The final spectra were smoothed using the adjacent averaging method with a 10-point window.

### Charge transport simulations

The ideal B-form and A-form structures were generated with the Nucleic Acid Builder software package^51^. *Ab initio* calculations were carried out with Gaussian 09 (ref. 47) Two sets of strands for both B-form and A-form DNA were considered—one with the sugar-phosphate backbone included and the other with the backbone deleted and the bases terminated with hydrogen atoms. For the strands with the backbone included, sodium counterions were used to neutralize the negatively charged phosphate groups. The sodium positions around the phosphate groups were found by relaxing the sodium counterions in a three-base single strand using the B3LYP/6-31G(d) exchange-correlation functional and basis set^52^,^53^,^54^,^55^. In the next step, calculations were carried out on the entire strand to obtain the Hamiltonian and overlap matrices *H*_0_ and *S*_0_ using B3LYP/6-31G(d,p). A unitary transformation based on the Löwdin transformation^56^,^57^ was implemented to convert *H*_0_ into a Hamiltonian with an orthogonal basis, *H*. The transmission probability was then calculated within the Landauer-Büttiker framework^48^. In the coherent limit, the transmission is calculated using,


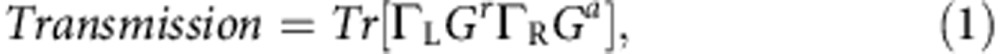


where Γ_L_ and Γ_R_ represent coupling to the left and right contacts, and *G*^*r*^ and *G*^*a*^ are the retarded and advance Green's functions, respectively. See the Supplementary Methods section for details.

## Additional information

**How to cite this article:** Artés, J. M. *et al*. Conformational gating of DNA conductance. *Nat. Commun.* 6:8870 doi: 10.1038/ncomms9870 (2015).

## Supplementary Material

Supplementary Information Supplementary Figures 1-13, Supplementary Tables 1-4, Supplementary Methods and Supplementary References
