# Supplementary material for: Conformational gating of DNA conductance
Source: Nat Commun. 2015 Dec 9;6:8870. doi: 10.1038/ncomms9870 (PMC4682165; doi:10.1038/ncomms9870)
Supplement: Supplementary Information — Supplementary Figures 1-13, Supplementary Tables 1-4, Supplementary Methods and Supplementary References [file ncomms9870-s1.pdf]

Supplementary Figures

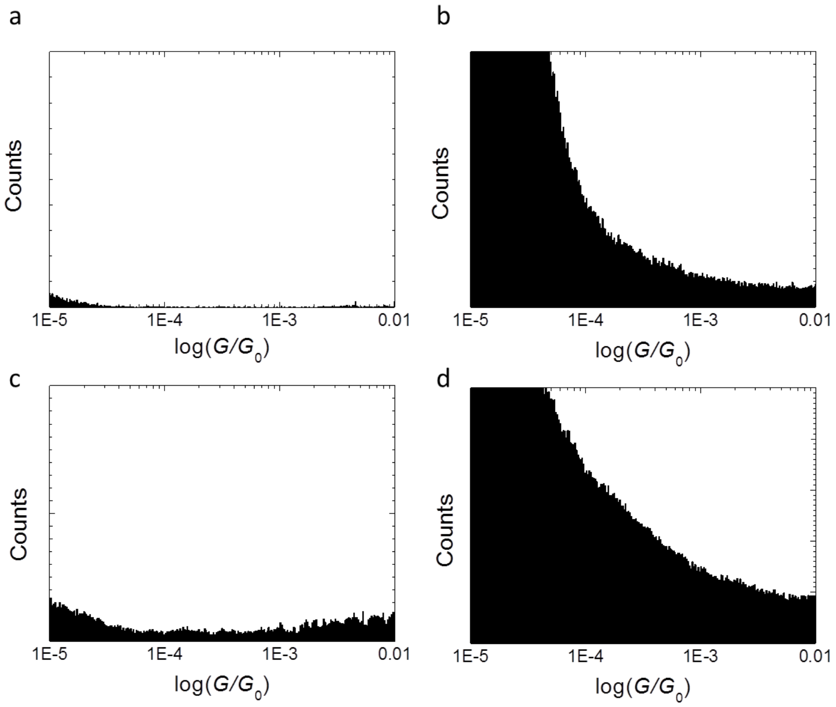

Supplementary Figure 1. Control experiments in 80% ethanol and in solutions containing non-functionalized dsDNA. Logarithmic conductance histograms obtained from blank experiments in 80% ethanol solution (a) including curves with some features identified by the selection algorithm and (b) including all curves. (c) and (d) correspond to control experiments of solutions containing 11-mer dsDNA without linkers at 5’ and 3’ ends, and using the same selection criteria.

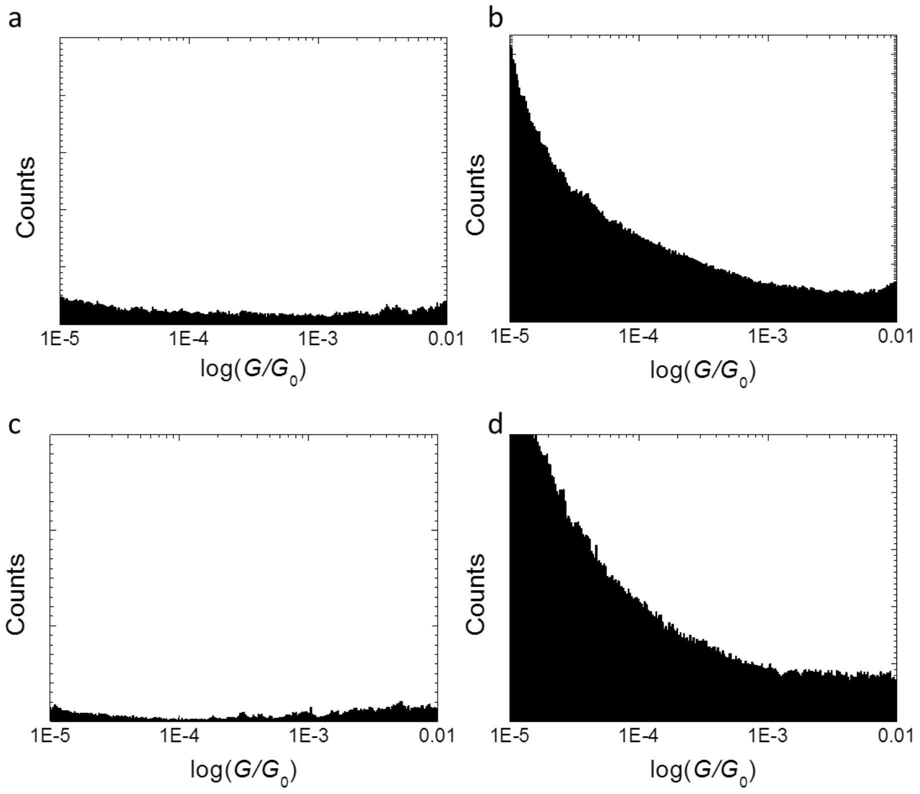

Supplementary Figure 2. Control experiments in solutions containing functionalized single-stranded 11-mer (ss)DNA. Logarithmic conductance histograms obtained from control experiments in solutions containing diamine-functionalized ssDNA (a) including curves with some features (b) including all the curves. (c) and (d) correspond to control experiments of solutions containing thiol-functionalized ssDNA.

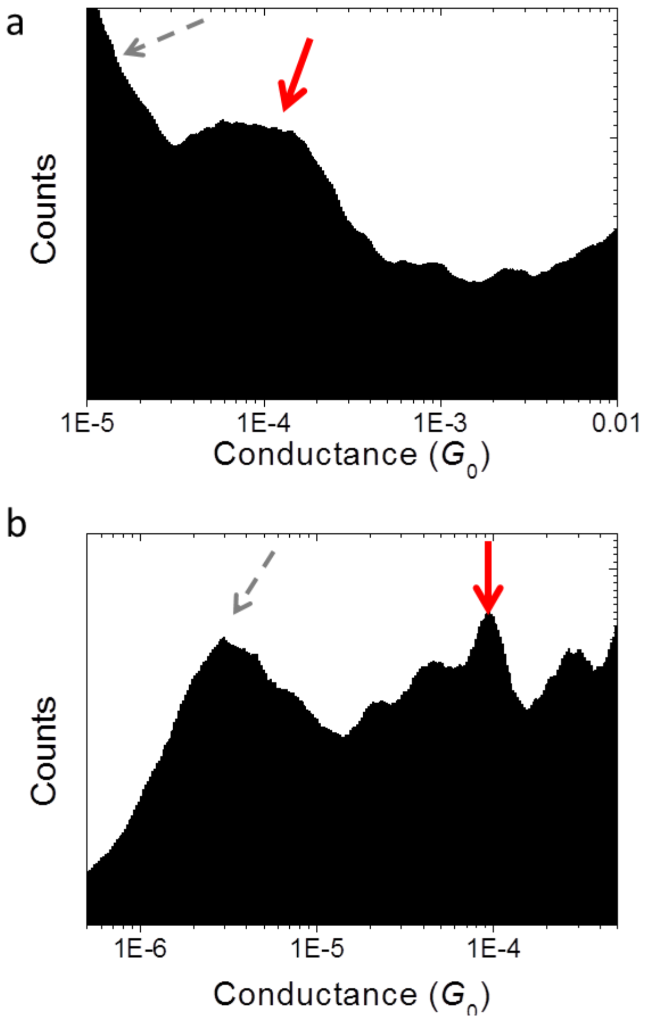

Supplementary Figure 3. Conductance histograms for the 11-mer dsDNA in buffer with different amplifiers and bias voltages indicated below. Dashed arrow indicates the lower limit (current amplifier noise level) while solid red arrow indicates the conductance peak obtained. (a) 10nA/V 50 mV bias and (b) 1 nA/V 10mV bias. Note that no other conductance peaks are present in the conductance ranges studied.

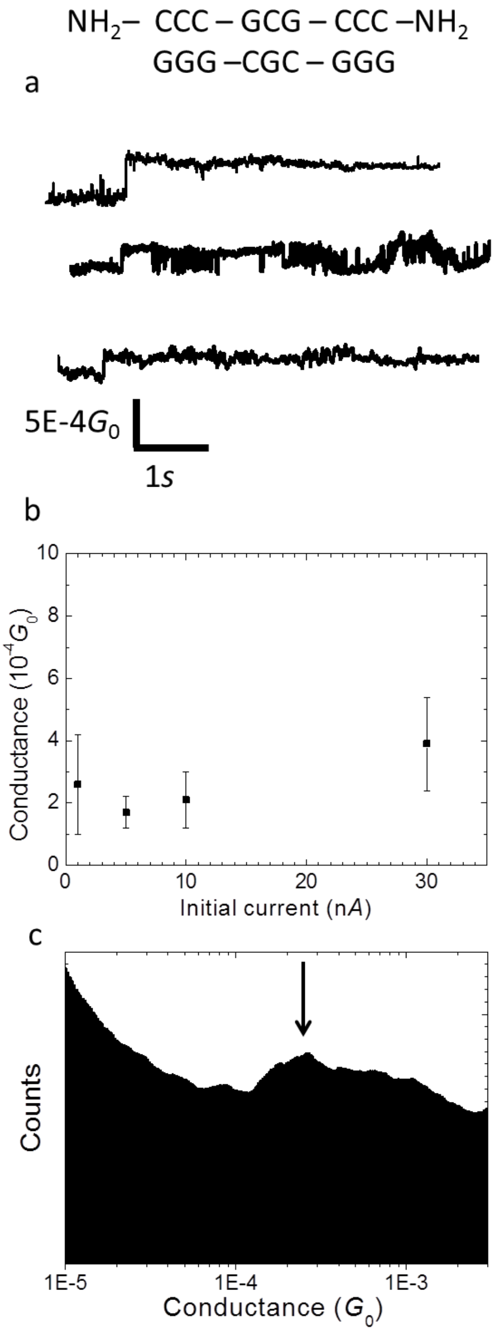

Supplementary Figure 4. Conductance from spontaneous formation of dsDNA junctions. (a) Conductance-time example recordings obtained from the spontaneous formation[1] of single molecule 9-mer dsDNA junctions in buffer solution. (b) Average conductance obtained from 10 traces similar to the ones shown in (a) at different initial current setpoint. Error bars are the standard deviations. The conductance histogram obtained by the STM-break junction method for the same molecule is shown in (c) for comparison.

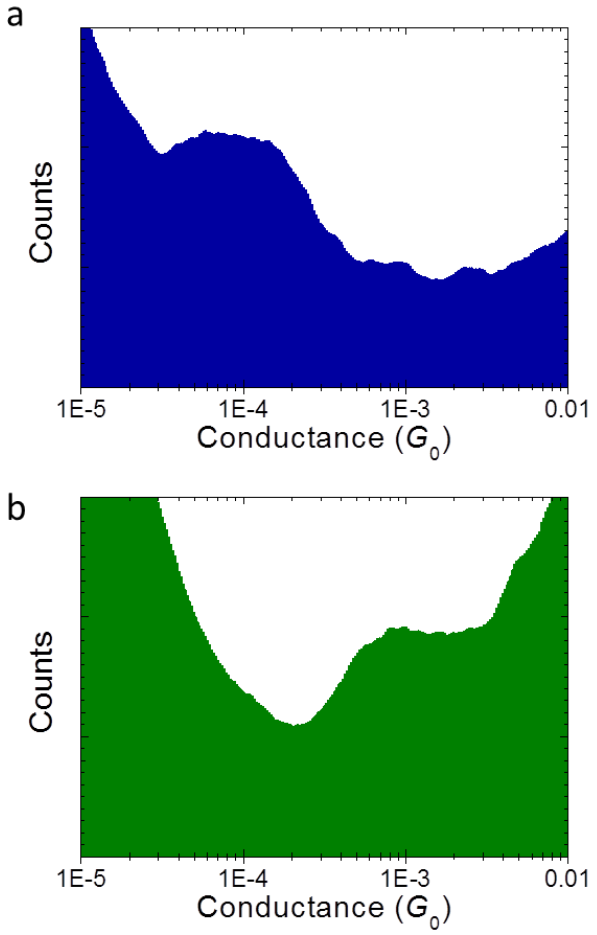

Supplementary Figure 5. Logarithmic conductance histograms obtained from thousands of current vs. distance traces for 11-mer B-form dsDNA in buffer (a) and A-form dsDNA in ethanol solution (b).

a

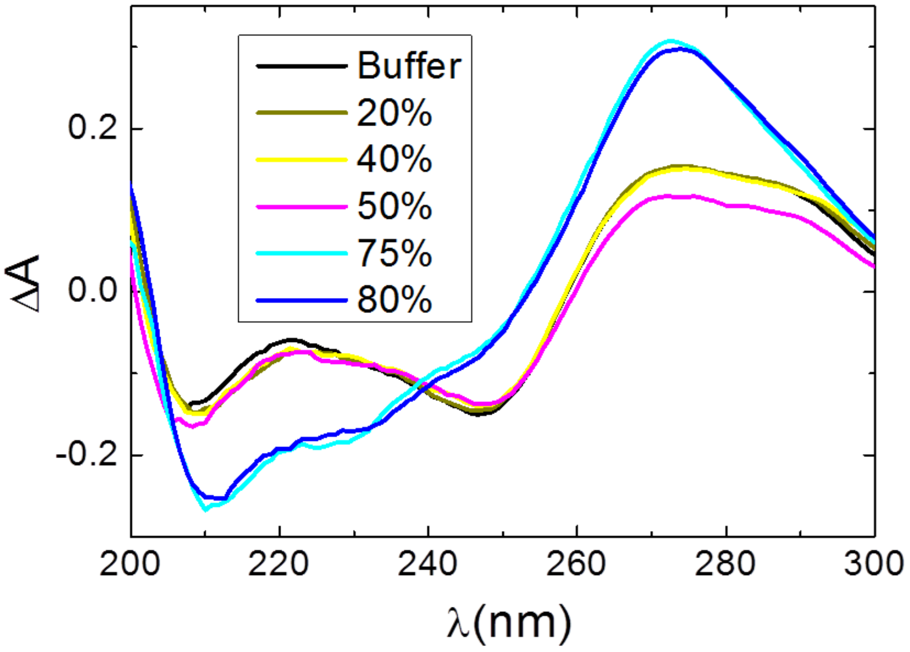

b

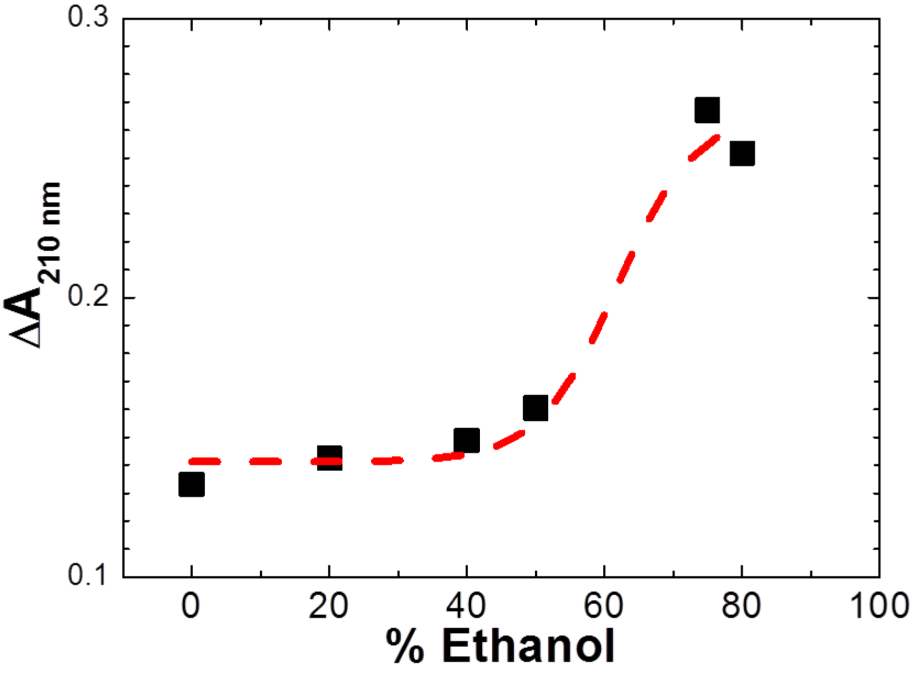

Supplementary Figure 6. Circular dichroism spectra for 11-mer dsDNA in 10mM buffer solutions with increasing ethanol concentration (a). The differential absorbance at 210 nm was used to monitor the transition from B to A-form dsDNA (b), as previously reported[2].

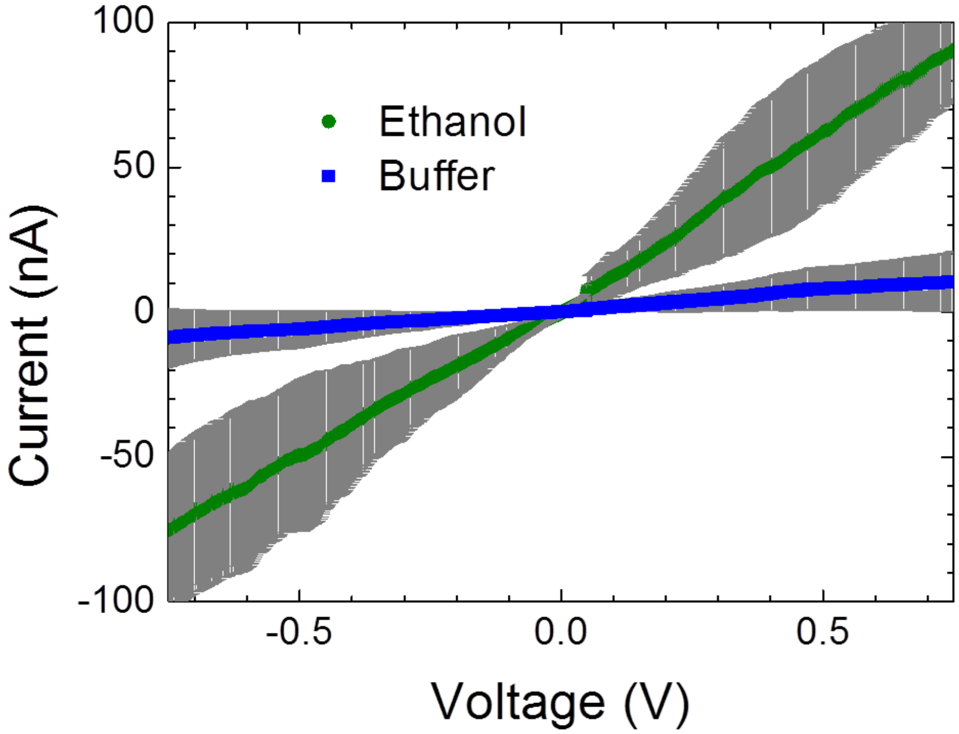

Supplementary Figure 7. Current vs. voltage average results obtained from experiments performed with 9-mer amino-functionalized dsDNA, using the approach described in ref. 3. Experiments in buffer are shown in blue while experiments performed in 80% ethanol are shown in green. Error bars represent the standard deviation and are shown in gray (N=20 *I-V* curves for each condition).

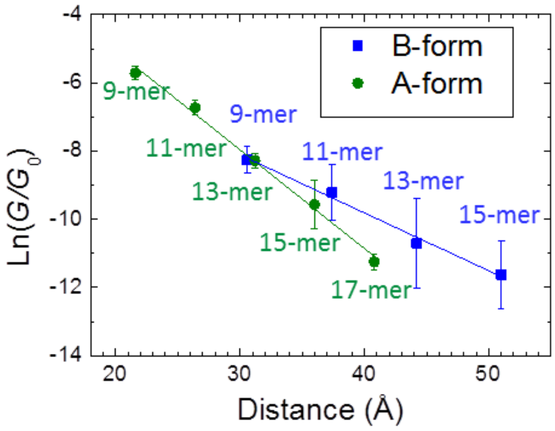

Supplementary Figure 8. Logarithmic conductance vs. distance plot of the 9-mer through the 17-mer. Error bars are the standard deviations of the normalized conductance values obtained from the histograms. Lines are linear regressions from which distance decay factors can be obtained. Values of 0.18 Å^{-1} and 0.33 Å^{-1} were obtained for the B-form and A-form, respectively. Error bars are the standard error of the mean from the average conductance, obtained from N=3 histograms in each case.

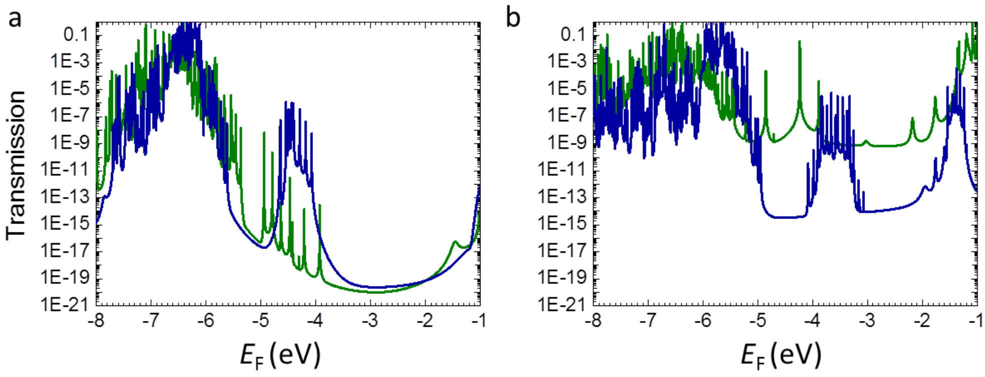

Supplementary Figure 9. Calculated transmission probability for A-form (green) and B-form (blue) dsDNA in the case that (a) the backbone is not included in the calculation, and (b) the case when it is included. Without the backbone, (a), the B-form DNA has a higher transmission probability around the HOMO level. When the backbone is included, (b), the transmission of B-form DNA increases by several orders of magnitude in the HOMO-LUMO gap, while the transmission of A-form DNA increases by approximately 10 orders of magnitude. And in some energy windows, the transmission of A-form surpasses that of B-form, demonstrating the importance of including the backbone in the transport models for these molecules. The data in (b) is the same as in Fig. 4(c) and is included here for clarity.

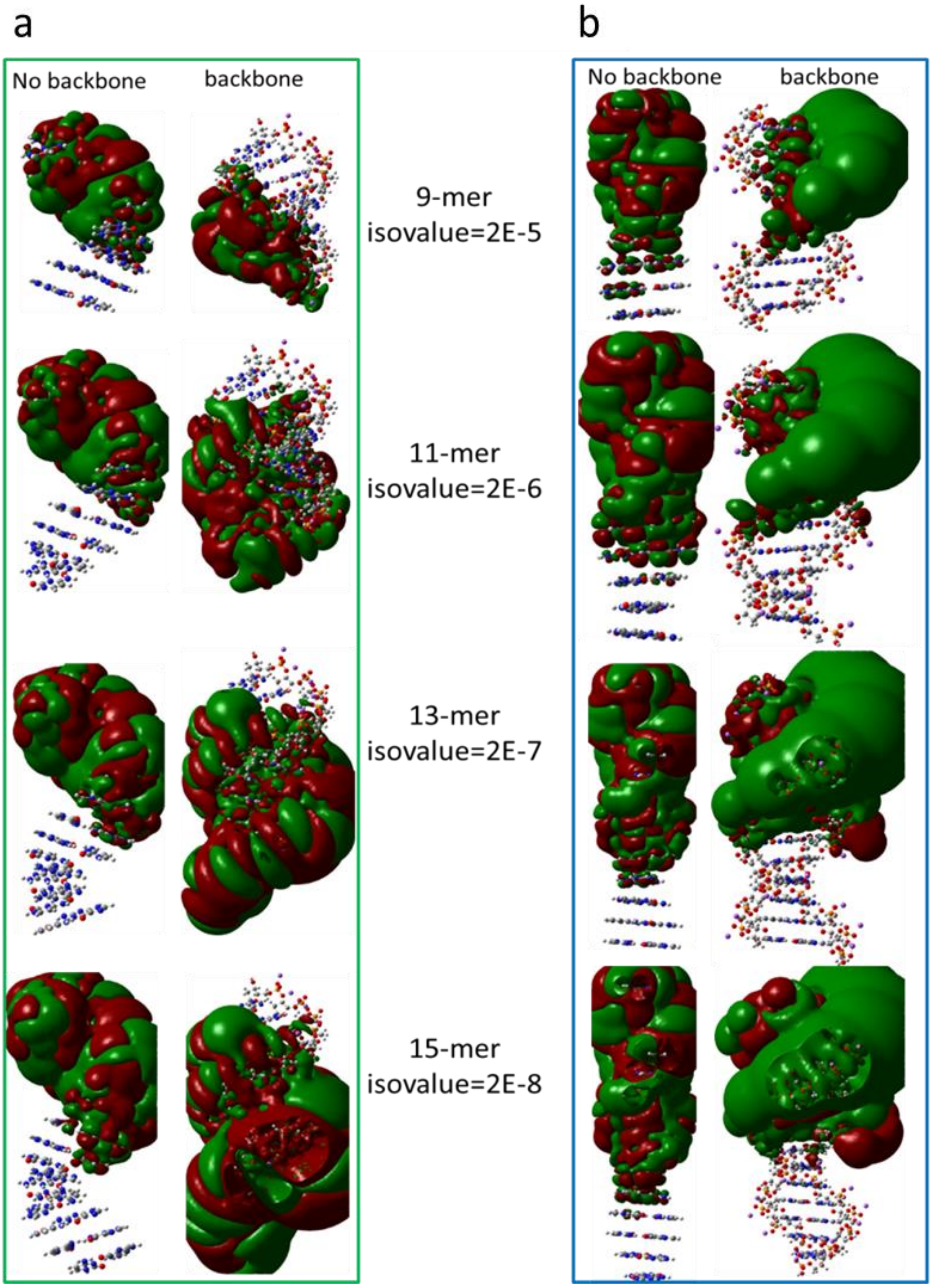

Supplementary Figure 10. 3D isosurface of the HOMO orbital on the oligonucleotide structures for A-form (a) and B-form (b) dsDNA, including (right) and not including (left) the backbone. Different isovalues (indicated) were used for the different lengths. The HOMO extends through nearly the entire molecule in the A-form when the backbone is included in the model, but not in any of the others cases for all the lengths studied.

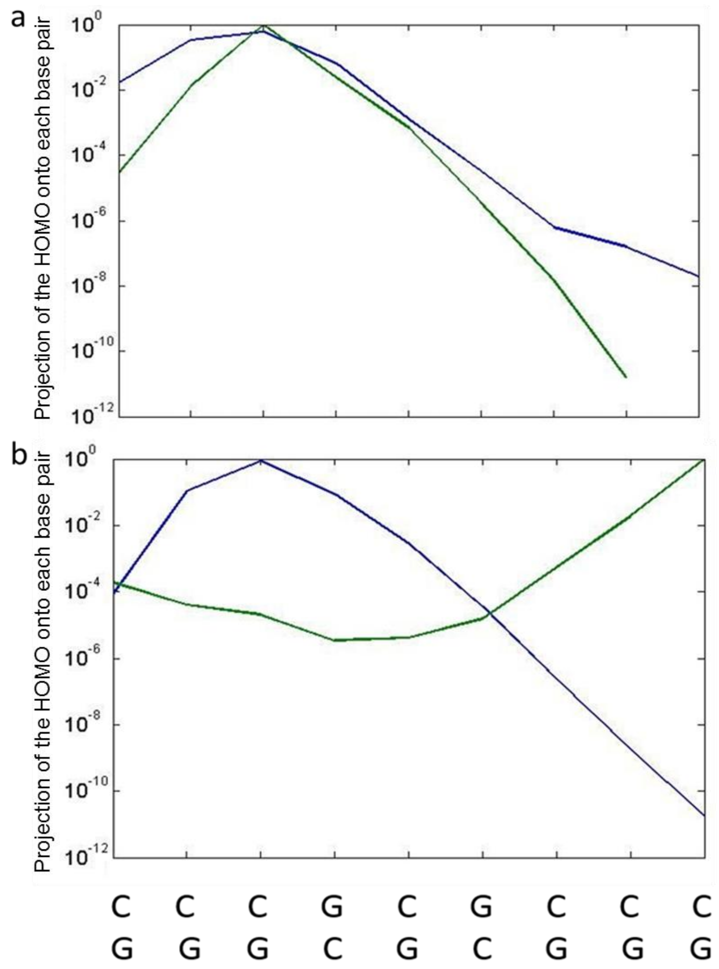

Supplementary Figure 11. Projection of the HOMO level onto each of the base pairs in the 9-mer A-form (green) and B-form (blue) oligonucleotide (5'-CCCGCGCCC-3', plus complementary strand), when the backbone is not included (a), and when it is included (b). The contribution from each base pair is more uniform in the A-form with backbone (b) than without the backbone (a). Note that in A-form DNA without backbone, the contribution of the 9th base pair is negligible. Only when the backbone is included, the projection of the HOMO level onto each base pair is more uniform in the A-form when compared to the B-form.

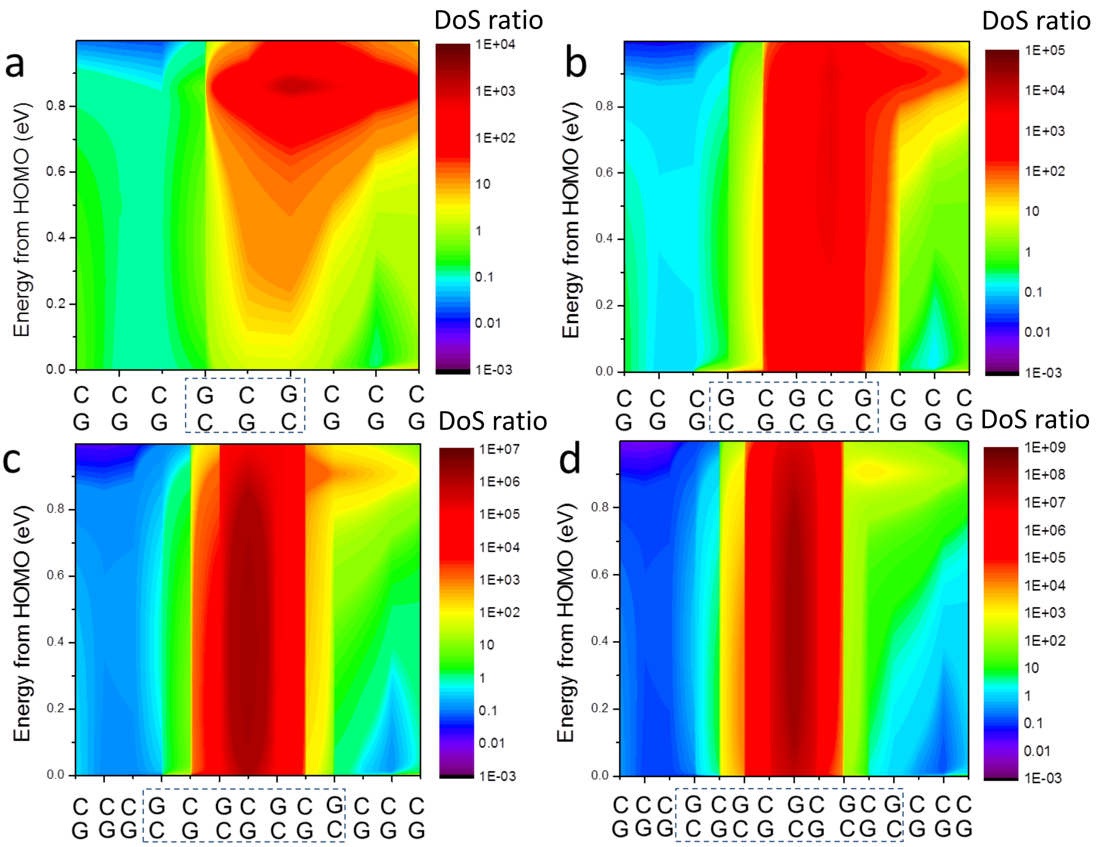

Supplementary Figure 12. 2-D representation of the ratio of the total density of states along the molecule between A-form and B-form for 1eV energy range in the HOMO-LUMO gap for 9-mer (a), 11-mer (b), 13-mer (c) and 15-mer (d). The DOS for the A-form in the GC bridge (see boxed region in the strands above) is larger for the A-form, leading to a larger conductance for the A-form. The x-axis represents the position along the strand and the y-axis represents the energy normalized to the HOMO level.

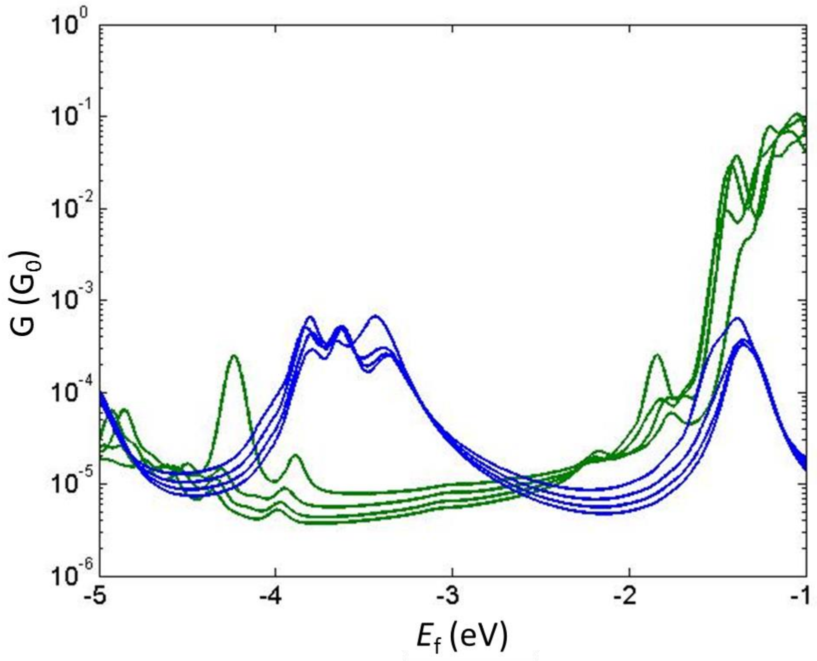

Supplementary Figure 13. Calculated conductance for B-form (blue) and A-form (green) dsDNA for different lengths (9-mer to 15-mer) with the backbone, and 10 meV decoherence rate. The conductance values of A-form DNA are larger than B-form in the gap region. The magnitude of conductance is closer to that from experiments when compared to the coherent case.

Supplementary Tables

Supplementary Table 1. Linkers used in the experiments

Functionalization (linker)	
Diamine	Dithiol
NH_2-(C_3H_6)-sequence-(C_3H_6)-NH_2<br>(3 Carbon spacer at both sides)	SH-(C_6H_{12})-sequence-(C_6H_{12})-SH<br>(6 Carbon spacer at both sides)

Supplementary Table 2. Sequences used in the experiments and Tm values.

Sequence:<br>Linker-5'--CCC-G(CG)$_N$-CCC-3'-Linker	n	Tm B-form (ºC)	Tm A-form (ºC)*
9-mer	1	55.6	55.9
11-mer	2	62.7	59.7
13-mer	3	67.3	62.2
15-mer	4	70.5	63.8
17-mer	5	72.7	75

**Tm values calculated using oligoanalyzer (IDT). A-form Tm was obtained from calculations considering RNA strands, as it is known to be in A-form*[S2]*.*

Supplementary Table 3. Coefficients for the projected HOMO along the 9-mer calculated without including the backbone.

	A-form		B-form	
Sequence	Strand 1	Strand 2*	Strand 1	Strand 2*
C	1.02E-07	2.68E-05	7.99E-05	1.60E-02
C	8.58E-06	1.30E-02	4.69E-04	3.33E-01
C	1.02E-03	9.68E-01	1.86E-03	6.03E-01
G	2.14E-02	2.38E-03	6.00E-02	3.55E-03
C	6.78E-04	1.29E-05	9.69E-04	3.00E-04
G	3.41E-06	6.73E-09	3.25E-05	1.25E-06
C	1.49E-08	1.62E-10	3.61E-07	2.64E-07
C	1.41E-11	2.06E-12	1.15E-09	1.56E-07
C	0.00	0.00	2.52E-10	1.87E-08

******Strand 2 is the complementary strand (sequence: GGGCGCGGG).*

Supplementary Table 4. Coefficients for the projected HOMO along the 9-mer calculated including the backbone for the two strands, for A-form and B-form, and along bases and backbone portions, as described below.

	A-form				B-form			
	Strand 1		Strand 2*		Strand 1		Strand 2*	
Sequence	Base	Backbone	Base	Backbone	Base	Backbone	Base	Backbone
C	1.50E-07	5.84E-06	1.23E-09	1.07E-12	5.19E-08	5.05E-10	4.09E-05	4.05E-05
C	4.73E-06	1.83E-04	1.47E-09	6.72E-09	3.17E-07	1.13E-08	9.82E-04	1.07E-01
C	9.17E-07	3.66E-05	2.10E-09	3.10E-08	8.03E-07	2.00E-08	1.54E-03	8.40E-01
G	1.16E-07	1.99E-05	3.55E-10	1.06E-07	1.60E-06	7.49E-09	5.41E-05	8.58E-02
C	5.54E-08	2.77E-06	1.02E-09	5.64E-07	6.07E-09	3.52E-09	2.83E-06	2.84E-03
G	3.51E-09	1.87E-06	1.30E-07	2.12E-06	1.99E-08	8.26E-09	3.73E-08	3.51E-05
C	9.91E-08	1.89E-06	9.26E-06	1.33E-05	1.45E-10	3.79E-09	2.52E-10	2.36E-07
C	8.98E-06	6.03E-06	1.64E-02	5.32E-04	4.67E-12	2.82E-10	2.75E-12	1.56E-09
C	7.71E-04	1.53E-05	9.83E-01	2.23E-03	0.00	9.88E-12	0.00	7.49E-12

Strand 2 is the complementary strand (sequence: GGGCGCGGG).

Supplementary Methods

Coherence-corrected hopping fitting

The conductance for each oligonucleotide was expressed as resistance (R = 1/G). The resistance of the oligonucleotide can be related to the number of hopping sites (N) by the following coherence-corrected hopping equation, as recently reported[4]:

$$R_{tot} = R_0 + \frac{h}{e^2}\frac{N-1}{1-2e^{-B(N-1)}\cos[C(N-1)+\Delta\varphi]}T_{GG}{}^{-1} \qquad \text{(equation 1)}$$

Where R_0 is the contact resistance and h is the Planck constant, *e* is electron charge, *N* is the number of hopping sites, T_{GG} is the average transmission from one guanine to an adjacent guanine, $B = \omega_0/\omega$ is the decay of coherence over distance (ω_0= 0.32 nm is the distance between bp for B-form, ω_0= 0.23 nm for the A-form, and ω is the coherent length), $C = (2\sqrt{2mE})/\hbar\omega_0$ (where m and E are the mass and energy of the holes), and $\Delta\varphi$ is the phase shift.

R_0 is taken to be $\frac{h}{e^2}$ but a value 50 times larger does not affect the analysis because the second term above dominates. Fitting to the above equation yields, B to be 0.04 and 0.06, C to be 2.8 and 2.7, and $\Delta\varphi$ to be -0.86 and -0.6, for A-form and B-form respectively. We used $\frac{h}{e^2}T_{GG}{}^{-1}$ to be 2.88131E6 and 1.93648E7 for A-form and B-form respectively. The number of hopping sites N is equal to the number of base pairs in each of the strands.

Projected HOMO calculations

While the HOMO distribution in Supplementary Figure 10 pictorially compares the distribution of the HOMO levels in A-form and B-form structures, we seek to more quantitatively understand the delocalization of the HOMO orbital in the strands. For this, we calculated the projected density due to the HOMO orbital, which allows us to quantitatively find the contribution due to each base and backbone along the strand. These results are presented in Supplementary Tables 3 and 4. The projected density was calculated using the following method. Two sets of strands for both B-form and A-form DNA were considered – one with the sugar-phosphate backbone included and the other with the backbone deleted and the bases terminated with hydrogen atoms.

When solving the Schrodinger equation, the $i-th$ molecular orbital, Ψ_i, is expanded using a set of basis functions $\{\phi_\alpha\}$,[5]

$\Psi_i = \sum_{\alpha=1}^{K} c_\alpha^i \phi_\alpha$ (equation 2)

where K is the number of basis functions.

The eigen value equation that is solved is:

$FC = SC\varepsilon$ (equation 3)

where F is the Fock matrix, $F_{\alpha\beta} = \langle \phi_\alpha | F | \phi_\beta \rangle$, and the S matrix represents the overlap between basis functions, $S_{\alpha\beta} = \langle \phi_\alpha | \phi_\beta \rangle$. C is a matrix of the expansion coefficients C_α^i. The $i-th$ molecular orbital Ψ_i is described by the $i-th$ column of C because the molecular orbitals $\{\Psi_i\}$ are orthonormal, $C^+SC = 1$.

To identify the contribution to all of the orbitals from individual parts within a molecule, we divide the system into N fragments (each fragment is a base pair and N is the number of base pairs),

$\Psi_i = \sum_{\mu=1}^{N} \sum_{\alpha\in\mu} c_\alpha^i \phi_\alpha$ (equation 4)

Correspondingly, the Fock matrix F, overlap matrix S, and molecular orbital coefficient matrix C can also be transformed into the fragmental format. To find out the orbital contribution from a particular fragment at the $i-th$ energy level, we expand the following expression,

$$C^{i+}SC^i = \sum_{\mu=1}^{N} \sum_{p\in\mu} C_p^{i+} S_{pp} C_p + \sum_{\mu\neq\upsilon}^{N} \sum_{p\in\mu,q\in\upsilon} C_p^{i+} S_{pq} C_q$$

$= \sum_{\mu=1}^{N} f_\mu + \sum_{\mu\neq\upsilon}^{N} o_{\mu\nu}$ (equation 5)

where $f_{\mu=} \sum_{p\in\mu} C_p^{i+} S_{pp} C_p$ is the contribution from the $\mu-th$ fragment and $o_{\mu\nu} = \sum_{p\in\mu,q\in\upsilon} C_p^{i+} S_{pq} C_q$ is the overlap between the $\mu-th$ and $\nu-th$ fragments.

Decoherence Calculations

It is worth noting that the fully-coherent transport models, while providing important insights into the experimental results, yield conductance values that are several orders of magnitude below the experimentally observed values. This fact is consistent with the

observation of the low β-values obtained experimentally, and suggests that some partially decoherent process dominates the transport. There are several possible approaches to overcome this deficiency. First, one can include decoherence phenomenologically in the Green's functions transport model. This approach increases the conductance to values which are significantly closer to the experimental value, and still maintains the higher conductance of A-form over B-form in the gap region (see Supplementary Figure 13), but the decoherence parameter is an open variable. The optimal ways to include decoherence in biological molecules is an open problem, with some recent advances based on time-dependent Green's function calculations to capture the role of the fluctuating environment[6, 7]. In this work, the phenomenological Büttiker-probes were used to capture the decoherence due to the environment[8, 9]. The effective transmission probability for an electron to transmit from the left (L) contact to the right (R) contact is $T_{eff} = T_{LR} + \sum_{i,j=1}^{N_b} T_{L,i} W_{ij}^{-1} T_{j,R}$, where N_b is the number of Büttiker probes and T_{ij} is the transmission between the ith site to the jth site in the phase-coherent case, $T_{ij} = \Gamma_i G^r \Gamma_j G^a$. In the above equation, Γ_i is the broadening matrix due to the coupling from the contacts. We set $\Gamma_{L(R)} = 100meV$ for the left (right) contact and $\Gamma_b = 10meV$ for each Büttiker probe. G^r is the retarded Green's function, $G^r = \frac{1}{E-H-\Sigma_L-\Sigma_R-\Sigma_B}$, where E is the energy, H is the Hamiltonian, $\Sigma_{L(R)}$ and Σ_B are the self-energy matrices from the left (right) contact and the Büttiker probes, which are determined by $\Sigma_{L(R)} = -i\Gamma_{L(R)/2}$ and $\Sigma_B = \sum_{i=1}^{N_b} -i\,\Gamma_b/2$. G^a is the advanced Green's function, $G^a = (G^r)^\dagger$. W^{-1} is the inverse of matrix W, the elements of which are $W_{ij} = [(1-R_{ii})\delta_{ij} - T_{ij}(1-\delta_{ij})]$, where R_{ii} is the reflection probability at probe i and $R_{ii} = 1 - \sum_{j\neq i}^{N} T_{ij}$. The zero-bias conductance as a function of Fermi energy is $G(E_f) = \frac{-2e^2}{h}\int dE T_{eff}(E)\frac{\partial f(E-E_f)}{\partial E}$, with E$f$ being the Fermi function and evaluated at 300K.

Supplementary References

1. Haiss W, Nichols RJ, van Zalinge H, Higgins SJ, Bethell D, Schiffrin DJ. Measurement of single molecule conductivity using the spontaneous formation of molecular wires. *Phys. Chem. Chem. Phys.* **6**, 4330-4337 (2004).

2. Kypr J, Kejnovská I, Renčiuk D, Vorlíčková M. Circular dichroism and conformational polymorphism of DNA. *Nucleic acids res.* **37**, 1713-1725 (2009).

3. Guo S, Hihath J, Díez-Pérez I, Tao N. Measurement and Statistical Analysis of Single-Molecule Current–Voltage Characteristics, Transition Voltage Spectroscopy, and Tunneling Barrier Height. *J. Am. Chem. Soc.* **133**, 19189-19197 (2011).

4. Xiang L, Palma JL, Bruot C, Mujica V, Ratner MA, Tao N. Intermediate tunnelling–hopping regime in DNA charge transport. *Nat. Chem.* **7**, 221-226 (2015).

5. Jensen F. *Introduction to Computational Chemistry*. Wiley (2013).

6. Gutiérrez R, Caetano R, Woiczikowski PB, Kubar T, Elstner M, Cuniberti G. Structural fluctuations and quantum transport through DNA molecular wires: a combined molecular dynamics and model Hamiltonian approach. *New J. Phys.* **12**, 023022+16 (2010).

7. Zhang Y, Yam C, Chen G. Dissipative time-dependent quantum transport theory: Quantum interference and phonon induced decoherence dynamics. *J.Chem. Phys.* **142**, 164101-164110 (2015).

8. Büttiker M. Four-Terminal Phase-Coherent Conductance. *Phys. Rev. Lett.* **57**, 1761-1764 (1986).

9. Buttiker M. Symmetry of electrical conduction. *IBM J. Res. Dev.* **32**, 317-334 (1988).
